# Supplementary material for: Rapid turnover of life-cycle-related genes in the brown algae
Source: Genome Biol. 2019 Feb 14;20:35. doi: 10.1186/s13059-019-1630-6 (PMC6374913; doi:10.1186/s13059-019-1630-6)
Supplement: Supplementary file 3 — Protein alignments, corresponding Gblocks html file and CDS sequences for the lineage conserved gametophyte-biased genes. (ZIP 2109 kb) [file 13059_2019_1630_MOESM3_ESM.zip › Con_GA_alignments/Ec-12_008070.aa_ali.fasta-gb.htm]

Ec-12\_008070.aa.fasta.trans.aa\_ali.fasta


## Gblocks 0.91b Results

Processed file: **Ec-12\_008070.aa.fasta.trans.aa\_ali.fasta**  
Number of sequences: **2**  
Alignment assumed to be: **Protein**  
New number of positions: **365** (selected positions are underlined in blue)

```
                         10        20        30        40        50        60
                 =========+=========+=========+=========+=========+=========+
Ec-12_008070     MLNDRLVAFPSSPSRSKAEGDIAVAEEDMILQPQQGTGVTPAGGPKQDDGSPVAEAECPP
MSTRG.50652.1    -----------------------------------ATKVSAAAAAEAEATNETALPAQSP
                                                                      #######


                         70        80        90       100       110       120
                 =========+=========+=========+=========+=========+=========+
Ec-12_008070     SRWIMVEPWSSLLLLSDHAREGRDDEILQDRKLAEALVGSREEQQAILQDVTRTRTDSPV
MSTRG.50652.1    SSWASFEPWNSLLLG-GHGRVGGDENEVQDRKLAEVRLDDGEQGRAILQDAARTRSDTAA
                 ##############   ###########################################


                        130       140       150       160       170       180
                 =========+=========+=========+=========+=========+=========+
Ec-12_008070     FGDEKARQWLQRSVSLYCRERGLRYSQGLTHLAAPWALITDPPCLDGFSYRCFRSFVDRF
MSTRG.50652.1    FRDPNVRQWLERSVSLFCRERNMHYTQGLTELAAPWALITEPPCLSSFSYRCFSSFMERF
                 ############################################################


                        190       200       210       220       230       240
                 =========+=========+=========+=========+=========+=========+
Ec-12_008070     APNIFSTAQEEAG------GG---TTLVVLEAHLVLLEQLLRFHDPALAAHMDKCFVSPA
MSTRG.50652.1    APNIFATAPDAGAAGKAEEGGQQRGAFVVLEAHLVLLDHLLRFHDPALAVHLDKCCAGPA
                 ########                   #################################


                        250       260       270       280       290       300
                 =========+=========+=========+=========+=========+=========+
Ec-12_008070     AYATPWFVTLFASQTPAPALLHLWRLMLEYGDPVLHQFLSLAWLVSNRLVLMETPPDDMP
MSTRG.50652.1    AYATSWFVTLFARQMPVPALLHLWQRLAEHGDPVLVQFLALAWLVGNRGILLNTPREDVL
                 ############################################################


                        310       320       330       340       350       360
                 =========+=========+=========+=========+=========+=========+
Ec-12_008070     ATVSGLRLRSAGVVDDVFRAAVALRESTPRLFCDVLRRACFAEDQEGDGGGSCEGRGAPA
MSTRG.50652.1    KTVTRLRLASADVVDRVFGDALALRESTPRLFCDMVRRACYSGGGTNAKAEPAAWTAAAA
                 ########################################                    


                        370       380       390       400       410       420
                 =========+=========+=========+=========+=========+=========+
Ec-12_008070     ----GGEQ--QSGPPAG----GPAAEP------RVSLLQGLRAEGVLSVDADQVAEILLA
MSTRG.50652.1    DSAAGLTVNSLPGRNAGSGEVGPVHSATKTRRFRVGLLRFLRATGVLTVDADQVAEILLA
                                                  ###########################


                        430       440       450       460       470       480
                 =========+=========+=========+=========+=========+=========+
Ec-12_008070     GPSSTAAPGTTTTTAAAAPTSPATGDDGWRSGSG-RIGGRFVLVDCRPP--APGGEGPAG
MSTRG.50652.1    QPS---AP------LASAVADPSDEEDGRCTDTAPRIRGRFVLVDCRPPREQEKGDGDGA
                 ###            #############       ##############           


                        490       500       510       520       530       540
                 =========+=========+=========+=========+=========+=========+
Ec-12_008070     GGMR----AG-GEAAGDG--RVMWRRIRPAESFGREAAAVAEVLREVGAGGD--------
MSTRG.50652.1    ASLSYGRSSGVREEFGVGGRHVIWRRINPAESFAKQVGAVSELLRESCGGGEGGEGEARK
                                      ##############################         


                        550       560       570       580       590       600
                 =========+=========+=========+=========+=========+=========+
Ec-12_008070     ------------------------------------------------------------
MSTRG.50652.1    SAPPLEAVSSDIDGAPLREGVFEEIALEHEVGVAIGSDGDGSNNKGAVETLTHVCFIGMG
                                                                             


                        610       620       630       640       650       660
                 =========+=========+=========+=========+=========+=========+
Ec-12_008070     -------------ALHAAATAEAALPP------------------------SRQL-----
MSTRG.50652.1    GLGNSSEWSDSRFASPAEAEARAAAPEFRLALVASLTCLTPHVCVLEGGFPALDQAFHRK
                              #############                                  


                        670       680       690       700       710       720
                 =========+=========+=========+=========+=========+=========+
Ec-12_008070     ------------------------------------------------------------
MSTRG.50652.1    GFEDARQNVSSVGAGNEKEPFAGAGHPPFAIGQESIDCPNGEVVATTTGSGGRDEARDEP
                                                                             


                        730       740       750       760       770       780
                 =========+=========+=========+=========+=========+=========+
Ec-12_008070     ----------------------------------------RLRRQPPSSGSR-----A--
MSTRG.50652.1    PRPDAAGGEGYLPADAVKTMPTTPAESSSPNPASPSGGGGGGDPAPPSSFSRGLERKASR
                                                                             


                        790       800       810       820       830       840
                 =========+=========+=========+=========+=========+=========+
Ec-12_008070     ------------------------------------------------------------
MSTRG.50652.1    LAGNRRISEPFRVYAAKSADDMAVGLRSLPIGASKSLEDAARVCNSLAKTNVRALGDSRL
                                                                             


                        850       860       870       880
                 =========+=========+=========+=========+====
Ec-12_008070     --------------------RRLAR------------------A
MSTRG.50652.1    SLSRSVSRLEQLNARMDGTARGFARHFGAGWLRGEEIGGSQVED
```

```
Parameters used
Minimum Number Of Sequences For A Conserved Position: 2
Minimum Number Of Sequences For A Flanking Position: 2
Maximum Number Of Contiguous Nonconserved Positions: 8
Minimum Length Of A Block: 10
Allowed Gap Positions: None
Use Similarity Matrices: Yes
```

```
Flank positions of the 8 selected block(s)
Flanks: [54  74]  [78  188]  [208  340]  [394  423]  [436  448]  [456  469]  [502  531]  [614  626]  

New number of positions in Ec-12_008070.aa.fasta.trans.aa_ali.fasta-gb:  365  (41% of the original 884 positions)
```
